# Prevalence, Pattern, and Outcome of Congenital Anomalies Admitted to a Neonatal Unit in a Low-Income Country—a Ten-Year Retrospective Study

**DOI:** 10.1007/s10995-023-03591-x

**Published:** 2023-02-28

**Authors:** Betty Anane-Fenin, Douglas Aninng Opoku, Lawrence Chauke

**Affiliations:** 1 Department of Obstetrics and Gynaecology, Faculty of Health Sciences, University of the Witwatersrand, Johannesburg, South Africa; 2 Department of Obstetrics and Gynaecology, Cape Coast Teaching Hospital, Cape Coast, Ghana; 3 School of Public Health, Kwame Nkrumah University of Science and Technology, Kumasi, Ghana; 4 Department of Obstetrics and Gynaecology, Charlotte Maxeke Johannesburg Academic Hospital, Johannesburg, South Africa

**Keywords:** Congenital anomalies, Congenital abnormalities, Birth defects, Neonates, Admission outcome

## Abstract

**Objective:**

The incidence of congenital abnormalities is highest in low-and-middle-income countries. However, the prevalence, spectrum, trends of neonatal congenital anomalies and their admission outcomes have not been well explored. This study was a 10 year retrospective hospital-based research in a low-income country to address the above.

**Methods:**

All infants hospitalized in the Special Care Baby Unit at the Cape Coast Teaching Hospital in Ghana, between 1st January 2010 and 31st December 2019, had their demographic, obstetric, and clinical data recorded.

**Results:**

Over the decade, 236 neonates with congenital abnormalities were admitted to the unit, accounting for 2.8% of total neonatal admissions and 8.6 per 1000 births. Mortality occurred in 33.2% of neonates with congenital abnormalities, corresponding to 4.6% of all neonatal deaths. Mortality was significantly associated with place of delivery and gravidity of more than five. The commonest anomalies were in the nervous system, particularly neural tube defects, followed by suspected chromosomal abnormalities and then cardiac defects. Neonates with cardiac defects had a higher chance of dying*.* Health center/clinic delivery proffered a better survival than hospital delivery, but this should be interpreted with caution.

**Conclusion:**

Neural tube defects were the most predominant anomalies; hence, intensification of preconception and antenatal folic acid supplementation is pivotal towards their reduction. Making prenatal screening for early detection of fetal anomalies an integral part of routine antenatal care is also essential. This research was conducted in a single center and did not include stillbirths and abortions so cannot give an accurate estimation of the number of congenital abnormalities in the population. A national registry of congenital anomalies is recommended.

## Significance

*What is already known?* It is known that congenital abnormalities or birth defects contribute significantly to under-five mortality and their incidence in LMICs forms about 94% of the global cases. Despite this high burden, there is a general lack of epidemiological data on the prevalence, pattern, and outcomes of congenital anomalies in Ghana and other LMICs.

*What this study adds?* This study provides a large database of data on neonatal congenital abnormalities and is the first to report on the prevalence and spectrum of congenital abnormalities in the Central Region of Ghana. The trend of neonatal abnormalities that warrant admission and their admission outcomes have also been explored for the first time in the country.

## Introduction

The World Health Organization (WHO) defines congenital disorders as “any potential pathological conditions arising before birth, whether evident at birth or manifesting later in life” (WHO). They are also referred to as birth defects, congenital malformations, congenital anomalies, or congenital disorders. The commonest of these defects are the structural (anatomic or morphological) abnormalities (Czeizel, 2005). Birth defects occur in about 6% total births worldwide (Christianson et al., 2006).

Congenital abnormalities in LMICs account for 94% of the total global number of congenital abnormalities and are among the top five causes of the under-five mortalities (Sitkin et al., 2015). Eighty percent (80%) of the global under-five mortality burden lies in Sub-Saharan Africa and Southern and Central Asia, (Christianson et al., 2006) with significant burden on the health systems. In the high-income countries, the implementation of prenatal counselling and screening, and the offer of termination of pregnancy for fetal anomalies (TOPFA) have helped to significantly reduce the birth prevalence and poor outcomes of infants with congenital anomalies. On the contrary, most low-income countries lack a structured system on prenatal screening and diagnosis. One major contributory factor is the paucity of data on the prevalence, spectrum, trends, and outcome of these anomalies which could have otherwise highlighted the problem as a major public health issue. Very few studies on congenital anomalies have been conducted in Ghana. One focused on malformations (except cardiac defects) in the general pediatric population and their outcomes, while another was based on autopsy findings in all age groups (nii-Amon-Kotei & Baffoe-Bonnie, 1991; Younn, 1963). A third one focused only on external structural defects at birth, (Nuertey et al., 2017) and one other, the spectrum of anomalies at admission without a report on admission outcomes (Ameyaw et al., 2017). Congenital anomalies are also not captured as a notifiable or reportable disease in Ghana; hence, no database or registry exists for them. Birth defect registers are helpful in establishing the incidence/prevalence, types, and trends of birth defects in a community or population, provides useful epidemiological data and an alert or warning sign for new teratogenic exposures in a community that may be accountable for new malformations or unusual presentations of previously established deformities. Furthermore, a registry allows researchers to discover potential causes of congenital defects, allowing for the planning of health service delivery and the design of preventative measures. It can also be used to assess the impact of prenatal screening and diagnostic procedures.

As a contribution to data on congenital abnormalities in LMICs, this study aims to narrow the knowledge gap on the prevalence and spectrum of neonatal congenital anomalies in Ghana, highlight for the first time, the trends of these anomalies and their neonatal admission outcomes.

## Materials and Methods

### Study Design, Site, and Population

This study was a retrospective descriptive research involving all neonates diagnosed with at least one congenital abnormality and admitted in the Special Care Infants Unit at the Cape Coast Teaching Hospital in the Central Region of Ghana between 1st January 2010 and 31st December 2019.

The 400-bed hospital serves as the primary tertiary referral centre for Ghana’s Central and Western Regions but also receives referrals from sections of the Ashanti and Greater Accra regions due to proximity. According to the 2010 Population and Housing Census, Central region has at least 2,201,863 inhabitants; Western Region 3,093,201; Ashanti Region 4,780,380; and Greater Accra Region 4,010,054. Cape Coast is the capital city of the region.

The Child Health Department has two in-patient wards—the neonatal unit which is preferably known as a special care infants’ unit (SCBU) as its facilities are not up to a standard neonatal intensive care unit as the highest level of mechanical ventilation offered is continuous positive airway pressure. The Paediatric ward admits older infants and stable neonates who have been de-escalated from the SCBU. Paediatric surgery services are limited in the hospital. A visiting pediatric surgeon runs an outpatient clinic and performs surgeries every two weeks. Neurosurgery and pediatric urology services also begun in CCTH after 2015. Because of the lack of a pediatric cardiologist and cardiac surgery services in the hospital, all cardiac cases are referred to the leading public tertiary hospital in the country, which is in the capital city in another region. Infants who need urgent care by the pediatric surgeon on days outside his visiting times, and infants who require intensive care services are also referred to the same hospital. All live infants with congenital anomalies, whether delivered in the hospital or referred in, are examined at the SCBU for a management plan. Neonatal autopsies are not routinely performed unless requested by the clinicians or parents of the infant.

### Exclusion Criteria

Stillbirths with congenital defects were excluded from this study. Neonates with more than 30% of missing data were also not included.

### Data Collection and Tools

All health records at the SCBU, including the Admissions and Discharges (A&D) book were used to obtain maternal and newborn data. Eligible neonates were entered into an Excel spreadsheet. Data available in the records include gestational age at delivery, place and mode of delivery, date of birth and age in days at the time of admission, sex, birthweight, diagnosis, and maternal’ information such as name, age, and parity. Information on maternal folic acid intake in the index pregnancy, chronic medical illnesses and whether the neonatal defects were diagnosed prenatally are not available in those records. The admission outcome (discharged alive, died, or referred) and the number of days spent on the ward were also recorded. Data was not collected withanonymity, but information was kept private and accessible to only the researchers.

### Definition of Terms

- **Preterm**: infants born before 37 weeks gestation,
- **Term**: infants delivered at gestational ages between 37 and the 40th week (the expected date of delivery)
- **Postdate**: pregnancy that is between 40 and 42 weeks gestation
- **Post-term**: pregnancy that has lasted beyond the 42nd week
- **Extremely low birthweight infant**: an infant that weighs < 1000 g at birth
- **Very low birthweight infant**: an infant that weights 1000–1499 g at birth
- **Low birthweight infant**: an infant that weighs 1500–2499 g at birth
- **Normal birthweight infant**: an infant that weighs 2500–3999 g at birth
- **High birthweight infant**: an infant that weighs ≥ 4000 g at birth
- **Health Center**: the first point of contact between the formal health delivery system and the client. It serves a population of about 20,000 and offers basic curative and preventive services. It is manned by a physician/medical assistant.
- **Clinic**: a health facility that does not meet the criteria for a hospital, as defined by the Health Facilities Regulatory Agency (Ghana Health Service, 1996; Health Facilities Regulatory Agency, 2022). It is manned by a medical doctor and provides a wider range of services than a health centre.
- **Hospital**: a bigger facility, serving a population of more than 100,000, and meets the requirements as stipulated by the Health Facilities Regulatory Agency (Ghana Health Service, 1996; Health Facilities Regulatory Agency, 2022).

### Categorization of Abnormalities Using the EUROCAT Subgroup Classification System

The International Classification of Diseases (ICD) coding system was not employed in this study. The abnormalities were categorized into 13 subclasses using a modified version of the EUROCAT subclassification system (Boyle et al., 2018). In this study, the subclass ‘limb’ was substituted with ‘musculoskeletal’. Osteogenesis imperfecta was also reclassified from ‘other abnormalities/ syndromes’ to ‘musculoskeletal anomalies’. All limb anomalies, such as polydactyly and talipes, are included in this grouping. The diagnosis ‘suspected chromosomal abnormalities’ was retained in the data collection, as found in the health records, because the suspicion was never confirmed due to the unavailability and/or the lack of knowledge about confirmatory testing (karyotyping) in the public health service. Neonates with malformations in more than one subgroup system but not perceived as syndromic or related to a recognised association or sequence were classified as ‘Multiple’. The category ‘Others’ included conjoint twins, Moebius syndrome and VACTERL (vertebral, anorectal, cardiac, tracheoesophageal, renal and limb abnormalities) association. The subclass ‘ear, neck and face’ was also incorporated into the subclasses ‘orofacial’, ‘multiple’ and ‘others’.

### Data Analysis

Data was entered into Microsoft Excel, cleaned, and exported to STATA 14 for analysis (College Station, TX, USA). Frequencies, proportions, percentages, mean with standard deviation, median, and charts were used to present descriptive data. The outcome variable was the status of the baby upon exiting from the SCBU or the Paediatric ward (discharged alive/referred or died). In a univariate analysis, Pearson chi-square was performed to examine the relationship between independent factors and mortality. At 95% confidence level, a p-value of 0.05 was considered statistically significant. The variables with significant p-values in the bivariate analysis were entered into a Cox regression analysis for adjusted hazard ratios. Kaplan–Meier survival graphs were created to compare survival among the various categories of variables that were associated to mortality.

## Results

### Background, Demographic, and Clinical Characteristics of Study Participants

During the ten-year period, 8346 neonates were admitted to the SCBU, 236 of whom had at least one congenital abnormality. A total of 1593 neonatal fatalities occurred. As a result, the prevalence of congenital anomalies at the SCBU was 2.8% (236/8346). Over the decade, total deliveries were about 27,320 (both live and stillbirths) in the hospital, giving the SCBU admissions rate in the hospital as 30.5% of total deliveries (305 per 1000 births). The mortality rate for newborns with congenital anomalies was 33.2% (n = 74/227), accounting for 4.6% (74 out of 1593) of all SCBU deaths over a ten-year period.

**Fig. 1:**
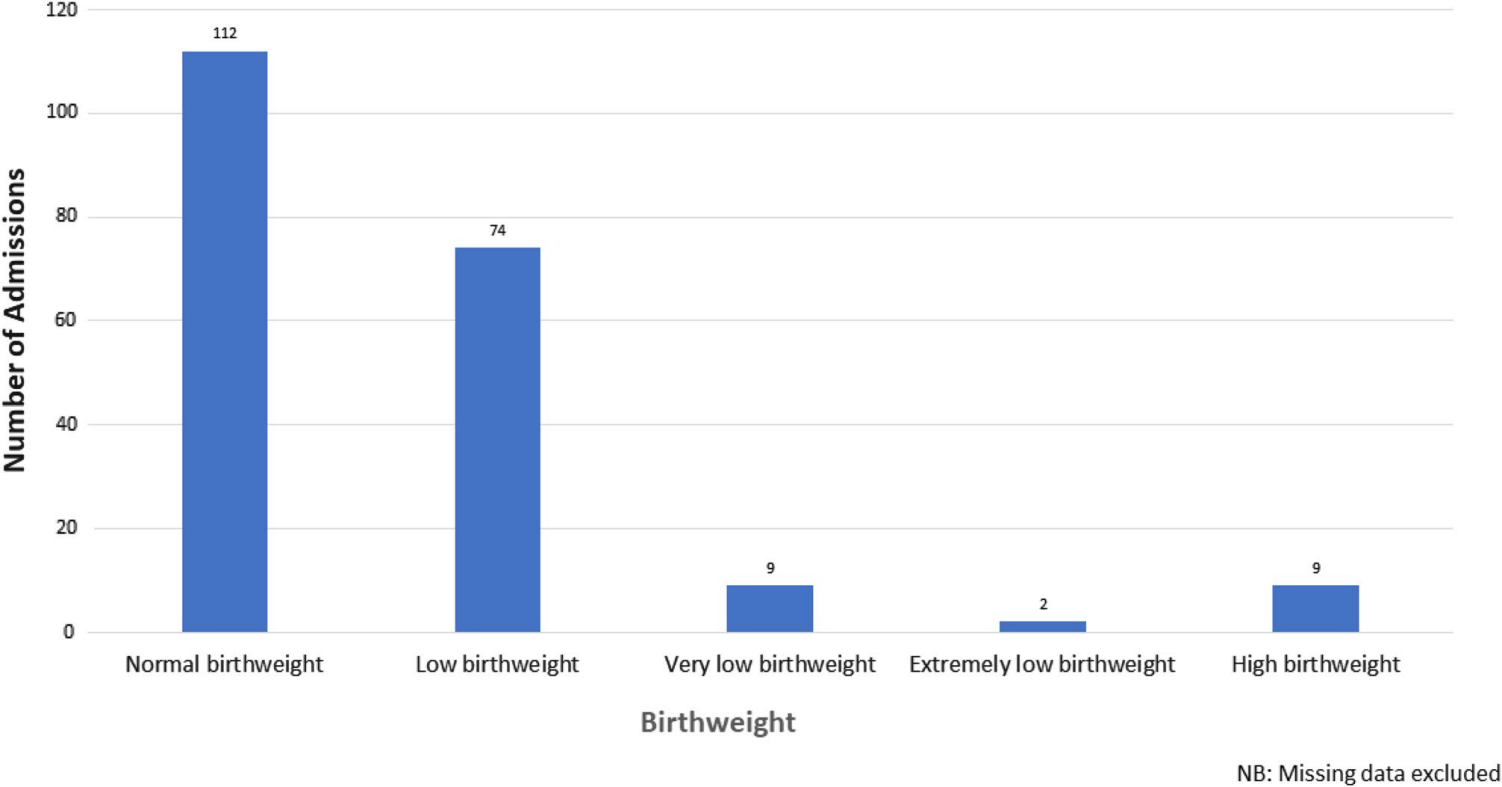
Birthweight of neonates

Table 1 shows the demographic characteristics of the women and the neonates. Age was not recorded in majority of the women and for those whose information were available, majority were between the 25–36 years, with the least being those who were over 35. No neonate’s name was counted more than once. Four neonates were identified as twins, but their mothers' identities were different, indicating that they were most likely from four different twin pregnancies. Figure 1 shows the birthweight categories of the neonates.

**Table 1:** Demographic characteristics of study participants

Variable	Frequency, N = 236	*Percentage, % [Range]
Age of mother (years) (n = 147)^a^		
< 25	53	36.1
25–35	79	53.7
> 35	15	10.2
Mean age (± SD)	26.8 (± 6.8)	[15–45]
Age of neonate (days) (n = 231)^b^		
0	154	66.7
1	38	16.5
2	8	3.5
3	9	3.9
4 +	22	9.5
Mean age (± SD)	0.9 (± 2.2)	[0–21]
Gender of neonate (n = 227)^c^		
Male	139	61.2
Female	87	87.4
Undetermined	1	0.4
Place of delivery (n = 226)^d^		
Clinic/Health center	26	11.5
Home	16	7.1
Hospital	184	81.4
Parity (n = 141)^e^		
Nulliparous	48	34.0
Primiparous	35	24.8
Multiparous	58	41.1
Median parity (± SD)	1.5 (± 1.5)	[0–6]
Gravidity (n = 110)^f^		
1–2	59	53.6
3–4	38	34.6
5 +	13	11.8
Median gravidity (± SD)	2.6 (± 1.7)	[1–8]

*May not add up to 100 because of rounding off errors

^a^89 (37.7%) missing value for age of mother

^b^5 (2.1%) missing values for age of neonate

^c^7 (3.0%) missing values for gender of neonate

^d^10 (4.2%) missing values for place of delivery

^e^95 (40.3%) missing data for parity; ^f^126 (53.4%) missing data for gravidity

Over half of the neonates were delivered vaginally. Majority of the newborns were admitted in SCBU for less than 7 days and nervous system anomalies were the most common congenital anomalies followed by suspected chromosomal anomalies and cardiac defects (Table 2).

**Table 2:** Mode of delivery, length of hospital stay, and diagnosis

Variable	Frequency, N = 236	*Percentage, % [Range]
Mode of delivery (n = 210)^a^		
Cesarean section	86	41.0
Vaginal delivery	124	59.0
Duration of stay (days) (n = 209)^b^		
< 7	145	69.4
7–13	40	19.1
14–20	9	4.3
> 20	15	7.2
Median duration of stay (± SD)	3.0 (± 7.2)	[1–43]
Birth defect category		
Abdominal wall	24	10.2
Cardiac	27	11.4
Digestive	21	8.9
Eye	1	0.4
Genital	3	1.3
Multiple	16	6.8
Musculoskeletal	19	8.1
Nervous system	54	22.9
Orofacial	15	6.4
Respiratory	1	0.4
Suspected chromosomal	39	16.5
Urinary tract	8	3.4
Others	8	3.4

*May not add up to 100 because of rounding off errors

^a^26 (11.0%) missing value for mode of delivery

^b^27 (11.4%) missing values for duration of stay

### Prevalence Trend Over the Study Period

Table 3 shows the trend in the prevalence of congenital abnormalities over the 10 year period, with the highest being recorded in 2014 to 2016 and 2019.

**Table 3:** Annual prevalence rates over the ten-year period

Year	Number of abnormalities	Total neonatal admissions	*Prevalence per 1000 admissions	Total births	*Prevalence per 1000 births
2010	12	1255	9.6	2307	5.2
2011	18	620	29.0	2104	8.6
2012	13	712	18.3	2635	4.9
2013	20	756	26.5	2656	7.5
2014	32	830	38.6	2618	12.2
2015	32	765	41.8	2854	11.2
2016	31	670	46.3	2904	10.7
2017	23	780	29.5	3055	7.5
2018	22	890	24.7	3160	7.0
2019	33	1068	30.9	3027	10.9
Total	236	8346		27,320	

*Rounded to one decimal place

### Outcome of Admission

**Fig. 2:**
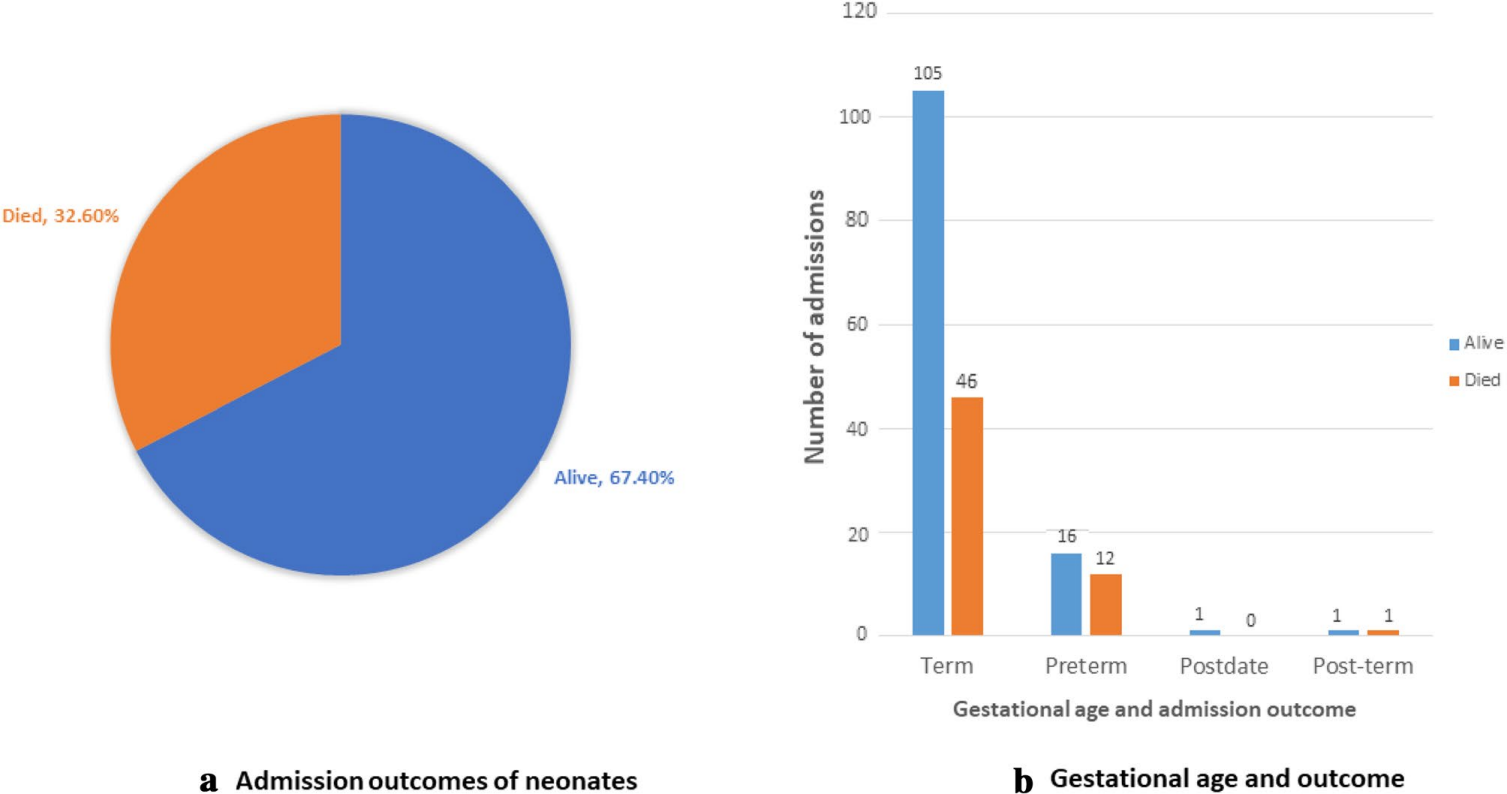
Gestational age and admission outcomes

Figure 2a, b show the overall outcome of the admissions and the outcome based on the gestational age at delivery. In decreasing order, the subclasses cardiac and multiple, and others had the highest mortalities over the decade (Table 4).

**Table 4:** Subclass mortality rate over the ten-year period

Subclass	Total number (N)	Survived (n^1^)	Died (n^2^)	Outcome not indicated (n^3^)	*Subclass mortality rate (%) (n^2^/N-n^3^ × 100)
Abdominal wall	24	20	2	2	9.1
Cardiac	27	10	16	1	61.5
Digestive	21	16	5	0	23.8
Genital	3	3	0	0	0.0
Musculoskeletal	19	10	7	2	41.2
Multiple	16	7	9	0	56.3
Nervous	54	37	15	2	28.8
Orofacial	15	14	1	0	6.7
Respiratory	1	1	0	0	0.0
Suspected Chromosomal	39	23	14	2	37.8
Urinary	8	7	1	0	12.5
Others/ syndrome**	9	4	5	0	55.6

*Rounded to one decimal place

**Includes the ‘Eye’ subclass (N = 1; n^2^ = 1)

The highest number of neonates who died with a diagnosis of congenital anomaly was observed in 2016 followed by 2015 and 2010 (Table 5).

**Table 5:** Contribution of congenital abnormalities to annual neonatal mortalities

Year	Total mortalities (N)	Mortalities among neonates with congenital anomalies (n)	*Proportion of mortalities due to congenital anomalies (%) (n/N × 100)
2010	101	5	5.0
2011	131	6	4.6
2012	131	3	2.3
2013	143	7	4.9
2014	186	6	3.2
2015	173	11	6.4
2016	179	12	6.7
2017	155	9	5.8
2018	176	6	3.4
2019	218	9	4.1

Missing data for outcome excluded from the calculation

*Rounded to one decimal place

### Factors Influencing Admission Outcomes of Neonates with Congenital Anomalies

**Fig. 3:**
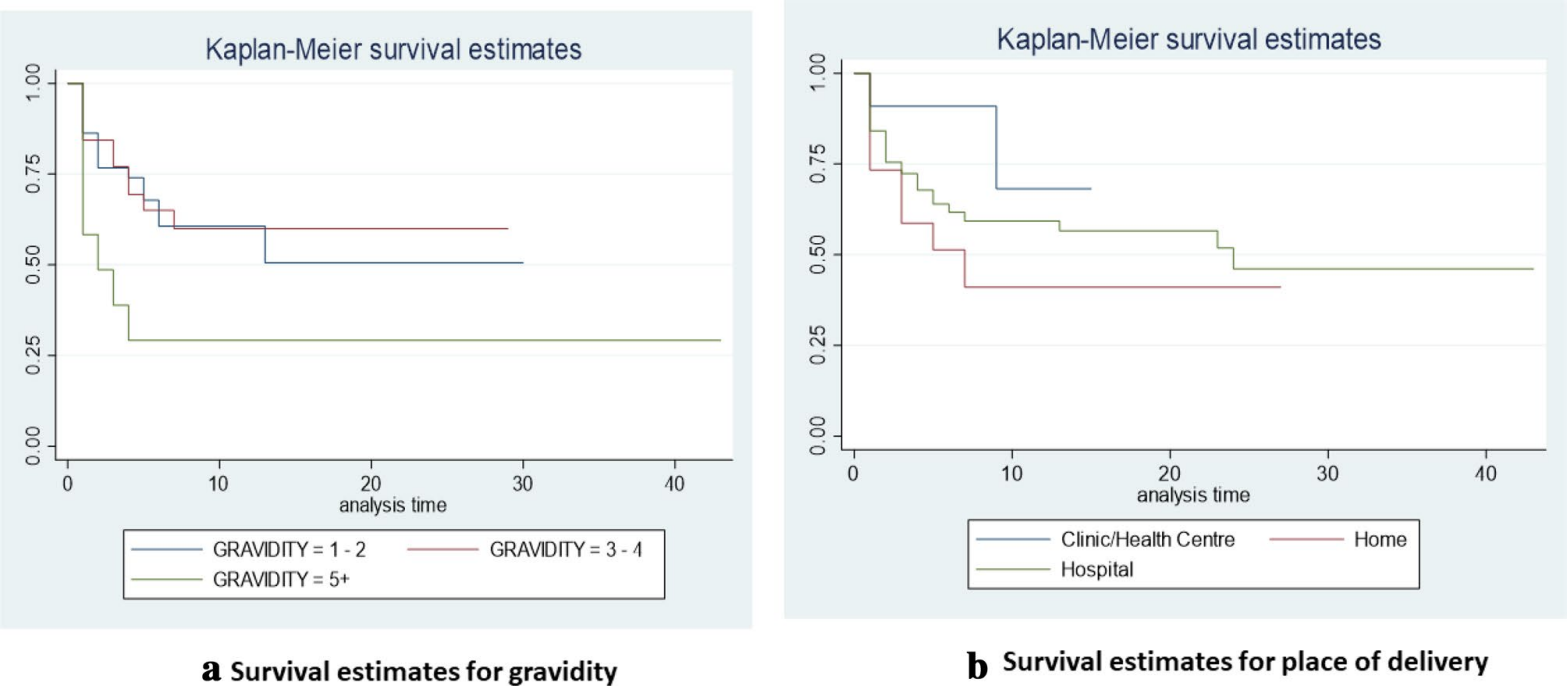
Kaplan–Meier survival estimates for gravidity and place of delivery

Table 6 shows that in this study cohort, hospital delivery and gravidity of more than five were significantly associated with death (AHR 2.61, 95%CI 2.61, 9.55, p = 0.001) and (AHR 3.29, 95%CI 1.20–8.97, p = 0.020), respectively. The survival estimates for gravidity and place of delivery are represented in Fig. 3. Delivery in a health center or a clinic have been captured together in the survival estimates because they both provide basic medical care.

**Table 6:** Factors influencing admission outcomes of neonates with congenital anomalies

	Crude hazard ratio (HR)		Adjusted hazard ratio (AHR)	
Variables	HR (95%CI)	P value	AHR (95%CI)	P value
Age of mother (years)				
< 25	1.00			
25–35	1.03 (0.55–1.93)	0.923		
> 35	0.66 (0.22–2.00)	0.464		
Age of neonates (days)				
0	1.00			
1	1.42 (0.79–2.54)	0.241		
2	0.75 (0.18–3.10)	0.694		
3	1.69	1.000		
4 +	1.40 (0.66–2.96)	0.380		
Gender of neonate				
Male	1.00			
Female	1.25 (0.78–1.99)	0.355		
Undetermined	1.01	1.000		
Place of delivery				
Clinic	1.00		1.00	
Health center	2.51	–	3.02	1.000
Home	7.32 (1.94–2.77)	< 0.001	1.06	–
Hospital	5.19 (1.62–1.66)	< 0.001	2.61 (7.12–9.55)	< 0.001
Parity				
Nulliparous	1.00			
Primiparous	1.78 (0.81–3.89)	0.150		
Multiparous	1.27 (0.61–2.64)	0.525		
Gravidity				
1–2	1.00		1.00	
3–4	0.96 (0.45–2.05)	0.915	1.06 (0.48–2.32)	0.889
5 +	2.61 (1.12–6.08)	0.027	3.29 (1.20–8.97)	0.020
Gestational age of neonate				
Postdate	1.00			
Post-term	–	–		
Preterm	0.31 (0.04–2.43)	0.267		
Term	0.24 (0.03–1.76)	0.161		
Weight of neonate				
Extremely low	4.81	1.000		
High	1.43 (0.44–4.71)	0.552		
Low	1.09 (0.63–1.91)	0.754		
Normal	1.00			
Very low	1.66 (0.59–4.72)	0.340		
Mode of delivery				
Cesarean delivery	1.00			
Vaginal delivery	0.87 (0.54–1.42)	0.585		
Birth defect category				
Abdominal wall	1.00		1.00	
Cardiac	5.15 (1.18–22.54)	0.030	2.70 (0.50–14.64)	0.251
Digestive	2.20 (0.43–11.36)	0.345	0.62 (0.06–7.52)	0.706
Eye	9.11 (0.82–100.88)	0.072	–	–
Genital	1.05	1.000	2.27	1.000
Multiple	5.74 (1.24–26.58)	0.025	2.75 (0.41–18.37)	0.296
Musculoskeletal	3.19 (0.66–15.41)	0.148	1.19 (0.20–7.08)	0.845
Nervous	2.50 (0.57–10.97)	0.223	2.78 (0.55–14.13)	0.217
Orofacial	0.78 (0.07–8.56)	0.836	2.38	1.000
Respiratory	1.04	1.000	7.61	1.000
Suspected chromosomal	3.01 (0.68–13.27)	0.146	1.96 (0.36–10.54)	0.434
Urinary	1.20 (0.11–13.22)	0.884	2.94	1.000
Others	3.21 (0.59–17.60)	0.179	1.75 (0.22–14.17)	0.599

## Discussion

This study is the first to report on the prevalence and spectrum of anomalies in the Central Region of Ghana and the first to highlight the trends of neonatal congenital abnormalities and their survival outcomes in the country. Congenital anomalies affect 6% of total births worldwide and 20–30 per 1000 livebirths (Christianson et al., 2006). In this study, congenital abnormalities were responsible for 2.8% (28 per 1000) of newborn admissions and 8.6 per 1000 births (both live and stillbirths). The former is comparable to the rates reported from Kenya and two other tertiary care hospitals in Gabon and Nigeria (Kamgaing, 2018; Nabea et al., 2017; Obu et al., 2012). However, it is significantly lower than the 7.22% recorded by Ameyaw and colleagues from another tertiary facility in Ghana (Ameyaw et al., 2017). The large disparity could be due to two factors: first, that hospital’s neonatal unit at the time of that study admitted infants up to three months of age, so their data is not just for neonates; and secondly, the hospital has a larger coverage area than CCTH because of its location in the Ashanti region, which is one of Ghana’s most populous regions, with nearly six million people as of 2021 (Ghana Statistical Service, 2020). Until recently, it also served as the only tertiary facility for the whole of the country’s northern belt. The use of varying study designs also account for the wide differences in the prevalence rates reported from some West African countries (Abbey et al., 2017; Akinmoladun et al., 2018; Ekwochi et al., 2018; Obu et al., 2012). When compared to high-income countries, the prevalence estimated from this study is much lower, (Boyle et al., 2018; Siddhisena et al., 2018) and this can be attributed to better prenatal and postnatal diagnostic systems, including autopsy in those settings.

### Trends in the Prevalence of Congenital Abnormalities Over the Ten-Year Period

Admissions due to congenital anomalies were at an all-time high from 2014 to 2016, and in 2019. The admissions were high for both per 1000 neonatal admissions and per 1000 births. In the year 2013, the hospital was upgraded from a secondary level facility and a regional hospital to a tertiary teaching hospital capacity. As a result, referrals increased, and this may explain the surge in congenital anomalies at the hospital between 2014 and 2016. Sanfaz and colleagues also report that their highest number of congenital abnormalities were seen in 2016 but no reason was attributed to it (Farag Sanfaz et al., 2019). The Zika virus outbreak in 2015 and 2016 increased congenital abnormalities in countries that were affected but the disease was never recorded in Ghana, making it an unlikely cause of the surge observed in 2014–2016. The reason for the drastic fall in the rate from 2017 to 2018 before rising again in 2019 is unclear.

### Spectrum of Congenital Abnormalities

Nervous system abnormalities were the most common, followed by suspected chromosomal abnormalities, cardiac, and abdominal wall abnormalities. In Kenya, Nigeria and Tanzania also, nervous system abnormalities dominate (Abbey et al., 2017; Akinmoladun et al., 2018; Kishimba et al., 2015; Silesh et al., 2021). A large percentage of the anomalies were neural tube defects. Contrarily, two systematic reviews conducted in LMICs found that musculoskeletal and cardiac malformations were the most common among newborns (Adane et al., 2020; Toobaie et al., 2019). Again, this is attributed to the different methodologies employed in the individual studies that were analysed. For example, some of the studies looked at external abnormalities in neonates while others included both prenatal and postnatal diagnoses and some focused on only those neonates that were admitted (Adane et al., 2020; Agot et al., 2020; El Koumi et al., 2013; Toobaie et al., 2019). In this study also, only the anomalies that required admission were included and most musculoskeletal defects such as isolated talipes and syndactyly do not often need admission.

Folic acid supplementation during pregnancy for the prevention of neural tube defects is an integral part of antenatal care in Ghana and it is most beneficial when initiated at least 3 months before conception, or soon after conception and continuing until at least the 12th week. However, late antenatal booking is a recognized maternal health concern in the Cape Coast metropolis (Amoako & Anto, 2021). The authors opine that the predominance of neural tube defects demonstrated in this study may be attributed to non-compliance of folic acid among pregnant women and late booking at the antenatal clinic.

### Outcomes of Neonatal Admissions for Congenital Abnormalities

About a third of neonates with congenital defects (33.2%) survived. This is similar to the observation made in an earlier study in another tertiary care facility in Ghana, which analyzed congenital malformations in the general pediatric population but excluded chromosomal and cardiac defects (nii-Amon-Kotei & Baffoe-Bonnie, 1991). In that study, the overall mortality rate was 33.5%, despite corrective surgery. Other studies have reported higher survival rates for neonates with congenital anomalies requiring admission, (Ajao & Adeoye, 2019; Siddhisena et al., 2018; Singh et al., 2021) and this is probably due to the severity of anomalies and the level of neonatal care, including prompt surgical intervention, but not related to gestational age or birthweight, as majority of the neonates in this study were also born at term and were of normal weight at delivery. The level of neonatal care is an important determinant in neonatal admission outcomes. Although great progress has been made towards preterm survival at the SCBU, despite its challenges with resources, it appears the major congenital abnormalities require more specialized services, including expertise and logistics for prompt intervention. This partly explains the lower survival rates in this study. An upgrade of the unit to a NICU and the attraction of permanent specialists/subspecialists in the various pediatric disciplines to the hospital will go a long way to overcome this challenge. Another reason could also be delayed interventions due to financial constraints of parents, considering the high cost of diagnostics and treatment for some of these anomalies.

#### Annual Mortality Rates from Congenital Abnormalities

Even though the prevalence rates were highest from 2014 to 2016 and 2019, the number of deaths due to congenital defects did not follow the trend. The highest mortalities were recorded in 2015 to 2017 (6.4%, 6.7% and 5.8% respectively); hence, the increasing number of admissions only resulted in greater mortalities in 2015 and 2016, and not 2014 and 2019. The reason for this trend is unclear although it suggests most of the cases during that period were either amenable to surgery or were just not fatal. Due to the unavailability of data on this from other studies, the finding cannot be appropriately situated in the national and global context. Further research is needed to ascertain the factors that accounted for the high mortality rates in those years.

#### Mortality Rates per Subclass Over the Study Period

The subclasses with the most mortalities were cardiac, multiple, and other anomalies/ syndromes. More than half of neonates with cardiac abnormalities (61.5%) died and all six that had a cyanotic heart disease, demised. This may support the view that there are more cardiac defects in Sub-Saharan Africa that are not amenable to surgery due to their severity (Higashi et al., 2013). Existing studies that found a high number of cardiac abnormalities did not provide data on whether mortality was also increased in this subclass (Ajao & Adeoye, 2019; Siddhisena et al., 2018; Singh et al., 2021). Perhaps early antenatal diagnosis with adequate delivery preparation including arrangements for prompt referral would have increased their chances of survival. The ultimate need for adequate pre- and post-operative care cannot be overemphasised as there is invaluable evidence that it influences survival significantly, with survival being highest in high-income countries and worst in low-income countries (Wright et al., 2021). There was no mortality in the genital and respiratory subclasses because they are frequently treatable.

### Factors Affecting Survival in Neonates with Congenital Abnormalities

The place of delivery and gravidity were found to be important predictors of death or survival in this study. When compared to abdominal wall defects, cardiac and multiple anomalies carried a greater risk of death, although the differences were not statistically significant. Meanwhile, prematurity, low birth weight and the presence of multiple anomalies have been the reported predictors of death or survival globally, with ethnicity and being an aboriginal having inconsistent results from different studies (Agha et al., 2006; Egbe et al., 2015; Glinianaia et al., 2020). Apart from multiple anomalies, the other factors were not found to be consistent with the findings of this study (Agha et al., 2006; Egbe et al., 2015; Glinianaia et al., 2020). Little information is available on the risk factors for mortality from congenital anomalies in African studies. Population-based studies conducted across the continent to generate epidemiologic data in this area may be necessary.

The present study shows that health center/clinic delivery has a better prognosis than hospital delivery. This data must be interpreted with caution, as these centres offer basic medical services, hence refer all complicated cases to the hospitals. It is likely that they may have earlier referred pregnant women with fetuses with congenital anomalies to the hospitals inutero, either with same diagnosis or a different antenatal diagnosis. As compared to those delivered at home, delivery in a health facility enhances the survival of neonates with congenital abnormalities. Home delivery was low in this study, and this is largely attributed to the progress Ghana has made in strengthening skilled birth attendants (SBA) as a strategy to lowering maternal and neonatal mortality. As of 2014, antenatal care and SBA utilization rates were 97% and 76%, respectively (Ameyaw et al., 2021; GSS et al., 2015). However, late or under-reporting of deformities by parents or families after home birth due to stigmatization could potentially be a factor to their poor survival (Avoke, 2010; Oti-Boadi, 2017).

This is the first study that shows gravidity (more than five) as a major risk factor for death in newborns with congenital abnormalities. Birth order greater than three (that is, parity more than three has been linked to the occurrence of fetal abnormalities, and maternal age and low birth weight, but not as a risk factor for mortality Farag Sanfaz et al., 2019; Gedamu et al., 2021). Whether socio-economic factors underlie the high gravidity, in which case the mortality risk could be better explained, is unclear. From the available data, it is difficult to explain this trend. To put this observation into the right perspective, it should be noted that gravidity and parity were missing in 53.4% and 40.3% of entries, respectively. Therefore, this data should be interpreted with caution.

The presence of multiple anomalies is also a risk factor for mortality, (Egbe et al., 2015) as each anomaly bears a certain degree of morbidity and mortality risk, which might lead to decreased coping abilities and, ultimately, death.

## Strengths

This study is the first research in the Central Region to document the prevalence, and spectrum of neonatal congenital abnormalities. The study also provides the largest database of neonates with congenital abnormalities that were admitted in Ghana, and it is the first in the country to provide data on the trends and admission outcomes of neonates with congenital abnormalities. The decade-long of data is another significant strength of this study. Although it was a hospital-based study, it gives a good reflection of congenital abnormalities that warranted admission and their admission outcomes in the region. This information is a useful epidemiological data from which more research may be conducted to better understand the causes, risk factors, management, and prevention of these anomalies in the Ghanaian population.

## Limitations

This research was a single-center study that did not include stillbirths or abortions so cannot give an accurate estimation of the number of congenital abnormalities in the population. Also, congenital anomalies that did not require admission were not included because data on them were not available; hence, the full spectrum of congenital anomalies seen in the hospital over the study period was not fully represented in this study. The few neonates that were admitted directly to the pediatric ward were also excluded since their captured data were far less than that documented at the SCBU; hence giving > 30% less of the data required for the study, but due to the small numbers, their exclusion does not affect the integrity of the findings of this study. The study was based entirely on previously collected data. Therefore, missing data could not be recovered. Again, detailed maternal factors, such as chronic medical illnesses, immunizations, folate supplements, and whether the congenital defects were diagnosed prenatally, could not be assessed.

Confirmation of chromosomal or genetic abnormalities was not possible as karyotyping and genetic testing are uncommon in Ghana's public health service. Admission outcomes could also be determined only up until the time of discharge, referral, or death. The survival rate after discharge or referral could not be determined.

## Conclusion

Nervous system anomalies (notably neural tube defects), suspected chromosomal abnormalities and cardiac defects are the commonest congenital anomalies among hospitalized neonates in the Cape Coast Teaching Hospital. Therefore, preconception and antenatal folic acid supplementation, and prenatal screening should be emphasized repeatedly at the antenatal clinics. Also, it is imperative to improve data collection in the hospital, build a national registry for congenital abnormalities and make them notifiable or reportable to acquire adequate data that can inform policies on their prevention and management.

## Data Availability

All data and material are available.
